# Global Transcriptome Profile of the Oleaginous Yeast *Saitozyma podzolica* DSM 27192 Cultivated in Glucose and Xylose

**DOI:** 10.3390/jof7090758

**Published:** 2021-09-15

**Authors:** Habibu Aliyu, Olga Gorte, Anke Neumann, Katrin Ochsenreither

**Affiliations:** Institute of Process Engineering in Life Science 2: Technical Biology, Karlsruhe Institute of Technology, 76131 Karlsruhe, Germany; olga.gorte@gmail.com (O.G.); anke.neumann@kit.edu (A.N.)

**Keywords:** glucose, microbial lipid, oleaginous yeast, RNA-seq, *Saitozyma podzolica* DSM 27192, sugar transporters, Weimberg pathway, xylose

## Abstract

Unlike conventional yeasts, several oleaginous yeasts, including *Saitozyma podzolica* DSM 27192, possess the innate ability to grow and produce biochemicals from plant-derived lignocellulosic components such as hexose and pentose sugars. To elucidate the genetic basis of *S. podzolica* growth and lipid production on glucose and xylose, we performed comparative temporal transcriptome analysis using RNA-seq method. Approximately 3.4 and 22.2% of the 10,670 expressed genes were differentially (FDR < 0.05, and log2FC > 1.5) expressed under batch and fed batch modes, respectively. Our analysis revealed that a higher number of sugar transporter genes were significantly overrepresented in xylose relative to glucose-grown cultures. Given the low homology between proteins encoded by most of these genes and those of the well-characterised transporters, it is plausible to conclude that *S. podzolica* possesses a cache of putatively novel sugar transporters. The analysis also suggests that *S. podzolica* potentially channels carbon flux from xylose via both the non-oxidative pentose phosphate and potentially via the first steps of the Weimberg pathways to yield xylonic acid. However, only the ATP citrate lyase (ACL) gene showed significant upregulation among the essential oleaginous pathway genes under nitrogen limitation in xylose compared to glucose cultivation. Combined, these findings pave the way toward the design of strategies or the engineering of efficient biomass hydrolysate utilization in *S. podzolica* for the production of various biochemicals.

## 1. Introduction

According to the forecast of the United Nations, the world’s population is expected to grow by ~20% to 9.7 billion people within the next 30 years [1]. The demand for energy and material resources will therefore also increase. To satisfy these requirements and to circumvent economic and environmental challenges, which are associated with reliance on fossil sources, renewable and sustainable energy sources need to be developed. Microbial lipids produced by oleaginous yeasts could contribute as one of the numerous alternative energy sources. Oleaginous yeasts are able to accumulate up to 70% of their cell dry mass to the storage lipids, mainly triacylglycerols [2]. As a carbon source, yeasts are able to metabolize a large spectrum of cheap and abundant raw materials, such as lignocellulosic plant biomass or waste from the agricultural and food industry, enabling carbon recycling within a biorefinery concept [3,4,5]. Moreover, yeast cell propagation proceeds with high duplication rates and the cultivation can be easily upscaled, in contrast to microalgae. In addition, yeast cultivation is independent of season, climate and location, which is highly advantageous compared to oil seeds [6,7]. However, the molecular and evolutionary mechanisms of oleaginicity in yeasts remain unclear. By phylogenetic comparisons of oleaginous yeasts, it becomes apparent that often the property of oleagenicity is not shared by distinct phylogenetic groups [8,9]. Thus, this characteristic cannot be identified by pedigree analyses and is still the subject of current research.

For lipid production by oleaginous yeasts, two pivotal conditions are essential to the cultivation process, namely, carbon excess and nutrient limitation, mostly nitrogen. The latter induces lipid synthesis and intracellular accumulation for energy storage [2]. Usually, a two-stage process is performed comprising a first nutrient-rich phase for cell mass formation and merging into a nitrogen deficient stage with excess carbon source [10,11,12,13]. To avoid the highly controversial “food-or-fuel” debate, renewable and sustainable carbon sources should be used for microbial lipid production. For that purpose, plant biomass is a potential candidate containing polymers of cellulose and hemicellulose with glucose and xylose as the most abundant constituent monosaccharides [14]. Therefore, the molecular transport and metabolism of these hexose and pentose sugars in oleaginous yeasts needs further clarification.

*Saitozyma podzolica* DSM 27192, isolated from peat bog soil in Karlsruhe, Germany [15], produces microbial lipid under nitrogen limitation and sugar acids, such as gluconic and xylonic acids, from glucose and xylose, respectively [10,15,16]. Characterization of the oleaginous profile of *S. podzolica* DSM 27192 showed, that the strain accumulates up to 31.8 and 27.53% lipid per dry biomass on glucose and xylose, respectively. A recent process optimisation revealed that both pH and temperature influence oil accumulation in *S. podzolica* DSM 27192 with ~40% increase in lipid productivity achieved under the optimised relative to the standard cultivation condition [10]. Despite these advances, including the improved sugar-uptake efficiency of ~91 and 92% for glucose and xylose, respectively, under fed-batch cultivation mode, the genetic basis of carbon flux partitioning between lipid and sugar acids syntheses remains unclear. In this work, we used an RNA-seq strategy to elucidate the gene expression dynamics of the oleaginous yeast *Saitozyma podzolica* DSM 27192 cultivated on glucose and xylose.

## 2. Materials and Methods

### 2.1. Yeast Strain and Experimental Set Up

The oleaginous yeast *Saitozyma podzolica* DSM 27192 was isolated from peat bog soil in the Black Forest of Germany [15]. The yeast is maintained as glycerol stock in −80 °C storage and reactivated on YM agar as previously described [10].

Cultivation of the yeast was performed in duplicates in 2.5 L benchtop bioreactors (Infors HT, Bottmingen, Switzerland; Minifors fermentor) containing 1.2 L mineral salt medium as previously described [10]. Briefly, the cultivation parameters were pH 4, 22.5 °C, 600 rpm and 1 vvm aeration rate for 144 h of process time. The first 48 h comprised a batch phase for biomass production. At the end of this phase, the concentration of glucose or xylose was ~10 g/L and the nitrogen (ammonia) concentration was nearly approaching zero. At 48 h, a continuous automatic feed supply was established to maintain glucose or xylose concentration constant at ~10 g/L for lipid production during the rest of the cultivation. Yeast growth and production were tracked by daily sampling and analysis of dry cell mass, carbon source and ammonia consumption and lipid and organic acid content as described in [10].

Briefly, 1 mL sample from each time point was collected and used to determine the cell dry weight (CDW) as well as the sugar, organic acid and ammonium concentrations. The sample was centrifuged at 20,000× *g* for 3 min. The pellets were washed using physiological saline and then centrifuged at 20,000× *g* for 3 min. CDW was determined by weighing the dried pellets (48 h at 60 °C) using a precision balance.

Glucose concentration was quantified by enzymatic assay using D-glucose test kit from R-Biopharm (Art. No. 10716251035, R-Biopharm AG, Darmstadt, Germany), according to the manufacturer’s protocol. 

Xylose and organic acid concentration were determined by analysing 10 µL (injection volume) of the supernatant obtained above using HPLC (Agilent 1100 Series, Agilent Technologies Deutschland GmbH, Böblingen, Germany) equipped with a Rezex ROA organic acid H+ (8%) guard column (8 µm, 50 mm × 7.8 mm) (Phenomenex Inc., Aschaffenburg, Germany) followed by a Rezex ROA organic acid H+ (8%) column (8 µm, 300 mm × 7.8 mm) (Art. No. 00H-0138-K0, Phenomenex Inc., Aschaffenburg, Germany) and a reversed-phase column Synergi™ 4 μm Fusion-RP 80 Å (150 mm × 4.6 mm) (Art. No. 00F-4424-E0, Phenomenex Inc., Aschaffenburg, Germany) used for organic acid analytics.

For xylose, 50 °C column temperature, 5 mM H_2_SO_4_ mobile phase with 0.5 mL/min flow rate were used, and the compound was detected using a refractive index detector (Agilent 1200 series, Agilent Technologies Deutschland GmbH, Böblingen, Germany). The supernatant was diluted with 20 mM KH_2_PO_4_ pH 2.5 prior to organic acids quantification. A gradient mobile phase comprising 20 mM KH_2_PO_4_ pH 2.5 and 100% methanol was used at 1 mL/min to separate the compounds. The oven column was set at 30 °C and the compounds were detected using a UV detector (220 nm).

Concentration of ammonium nitrogen was determined photometrically by modifying the assay volume to 300 μL per sample in microtiter plates using the Spectroquant kit (1.14752.0001, Merck KGaA, Darmstadt, Germany).

Lipids were first derivatized to fatty acid methyl esters (FAMEs) by direct transesterification of yeast cell mass as described by [15,17]. Approximately 20–30 mg freeze-dried cell mass was acid esterified using a two-phase system. The first phase comprised 0.5 mL 2 mg/mL heptadecanoic acid in hexane and 1.5 mL pure hexane. The second phase contained equal volumes of 2 mL 15% H_2_SO_4_ in methanol. A thermo-shaker (Universal Labortechnik, Leipzig, Germany) was used to incubate the mixture at 100 °C and 1000 rpm for 2 h. Samples were vortexed at 30 min intervals, and the reaction was stopped by placing the reaction tubes on ice for 10 min. The upper phase containing FAMEs was analysed by gas chromatography (GC) using the 6890N Network GC-System (Agilent Technologies Deutschland GmbH, Waldbronn, Germany) coupled with a DB-Wax column (30 m × 0.25 mm) (Art. No. 122–7032; Agilent Technologies Deutschland GmbH, Böblingen, Germany). Sample separation was done by using a temperature gradient of 40–250 °C at 8 °C/min. Final temperature was maintained at 250 °C for 10 min and the FAMEs were detected and quantified based on the RM3 FAME Mix standard (Art. No. 07256-1AMP, Sigma Aldrich, Taufkirchen, Germany).

### 2.2. RNA Isolation, Library Construction and Sequencing

To compare the transcriptome profiles of *Saitozyma podzolica* DSM 27192 under different carbon sources and over the course of the two growth phases, biomass was sampled at 22, 46, 70 and 120 h. The biomass was pelleted by centrifugation, snap-frozen with liquid nitrogen and stored at −80 °C until RNA extraction. Total RNA extraction and mRNA sequencing was performed at Microsynth AG (Balgach, Switzerland). Illumina-stranded TruSeq RNA libraries were prepared after poly(A) enrichment and sequenced based on Illumina NextSeq (2 × 150 bp as paired-end) sequencing chemistry.

### 2.3. RNA-Seq Data Analyses

Raw reads were evaluated with FastQC v0.11.9 [18] and low-quality reads filtered using fastp v0.20.1 [19] with default parameters. High-quality reads were aligned against the *Saitozyma podzolica* DSM 27192 draft genome sequence [20] using HISAT2 v2.1.0 [21]. The samtools v1.2 [22] sorted BAM files we used to perform reference guided transcriptome assembly using StringTie v1.3.4d [23,24] with ‘-e’, and ‘stringtie –merge’ with ‘-m 300 -c 0.5 -F 0.5 -f 0.05′ options. The resultant assembly was evaluated using gffcompare v0.10.6 [25]. To generate gene counts for differential gene expression analysis, we used prepDE.py (http://ccb.jhu.edu/software/stringtie/dl/prepDE.py, accessed on 20 August 2020). Differential gene expression (DGE) analysis was performed using DESeq2 [26] implemented in iDEP v9.2 [27] with gene count filter of counts per million (CPM) ≥ 0.5 in at least 2 libraries. The log2 fold change (log2FC) and false discovery rate thresholds were set at <0.05 and >1.5, respectively. The count data was transformed based on the DESeq2 [26] regularized logarithm transformation (rlog) method and principal component analysis (PCA) was computed and visualised using iDEP v9.2 [27]. Time course DGE analysis was performed using maSigPro package [28,29] in R v4.0.2 with default settings except number of cluster k set at 6 and *R*^2^ threshold of ≥0.7.

### 2.4. Functional Analysis of Expressed Genes

Stringtie assembled transcripts were processed further using TransDecoder v5.5.0 (https://github.com/TransDecoder/TransDecoder, accessed on 20 August 2020) to obtain putative protein sequences. The candidate protein sequences obtained with TransDecoder were subsequently compared with predicted proteins from the *Saitozyma podzolica* DSM 27192 draft genome sequence with the aid of cd-hit v4.8.1 [30] to compile a complete set of full-length protein sequences for all expressed genes. The protein sequences were annotated using pannzer2 [31], KofamKOALA [32], eggNOG [33] and InterProScan v5 [34], respectively. The functional enrichment analysis reported here is primarily based on gene ontology (GO) annotation, unless otherwise stated. To evaluate the functional implication of differential gene expression, we performed GO term enrichment analyses using WebGestalt 0.4.4 with default setting and to reduce GO term redundancy, affinity propagation based on apcluster was applied [35].

### 2.5. Phylogenetic Analysis of Sugar Transport Proteins

The predicted protein sequences associated with overexpressed sugar transporters were further annotated using BLASTp against the conserved domain database [36], the curated UniProtKB/Swiss-Prot [37] and transporter classification [38] databases as well as prediction of transmembrane helices using TMHMM Server v2.0 [39]. The proteins were aligned using PSI/TM-Coffee [40] with UniRef100 homology extension and transmembrane sequence type options. A phylogenetic tree was constructed using IQ-TREE v1.6.11 [41].

## 3. Results and Discussion

### 3.1. Overview of Yeast Cultivation and RNA-Seq

**Figure 1:**
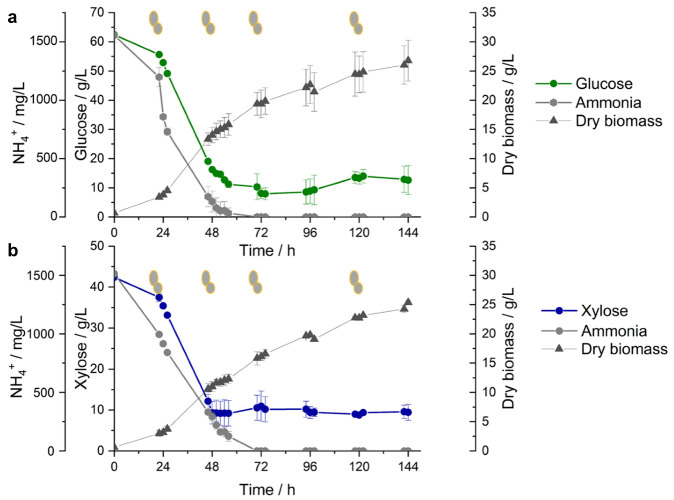
Overview of *Saitozyma podzolica* DSM 27192 cultivation on (**a**) glucose and (**b**) xylose. A batch-fed process was established at ~48 h for both sugars under nitrogen limitation. Grey cells indicate sampling points at which the yeast was harvested for RNA sequencing and subsequent analyses.

The oleaginous yeast *Saitozyma podzolica* DSM 27192, cultivated on glucose and xylose (Figure 1) was sampled for RNA-sequencing (RNA-seq) at 22, 48, 70 and 120 h with a view to evaluate the transcriptional response of the yeast during growth on the two sugars. For this study, we partitioned the cultivation into two stages. During the initial batch phase (~0–48 h) on glucose (Figure 1a), the yeast grew to a maximum cell mass of 14.03 ± 1.31 g/L at 48 h. At this time point, ~91.44% of ammonium was metabolised by the yeast. Cultivation of the yeast under batch mode on xylose followed the trend observed with glucose (Figure 1b). After 48 h growth on xylose, the yeast attains a maximum cell mass of 11.00 ± 0.85 g/L and metabolised ~80.65% of the nitrogen source. The second phase (~48–144 h) involved establishment of a continuous feeding at ~10 g/L sugars concentration under nitrogen limitation as previously described [10]. In both glucose and xylose cultures, ammonium was metabolised completely around 70 h (Figure 1a,b). The biomass increased steadily to a maximum of 26.78 ± 3.50 (Figure 1a) and 25.38 ± 0.25 g/L (Figure 1b) on glucose and xylose, respectively. The latter phase is characterised by accumulation of single-cell oil (SCO), a process triggered by nitrogen limitation, with the complete depletion of the nitrogen source at 70 h [9]. For instance, compared to sampling time point 46 h, lipid accumulation, estimated based on percent FAME per cell dry weight, increased by ~132 and 126% at 90 h and by ~209 and 195% at 144 h for glucose and xylose cultures, respectively [9].

**Figure 2:**
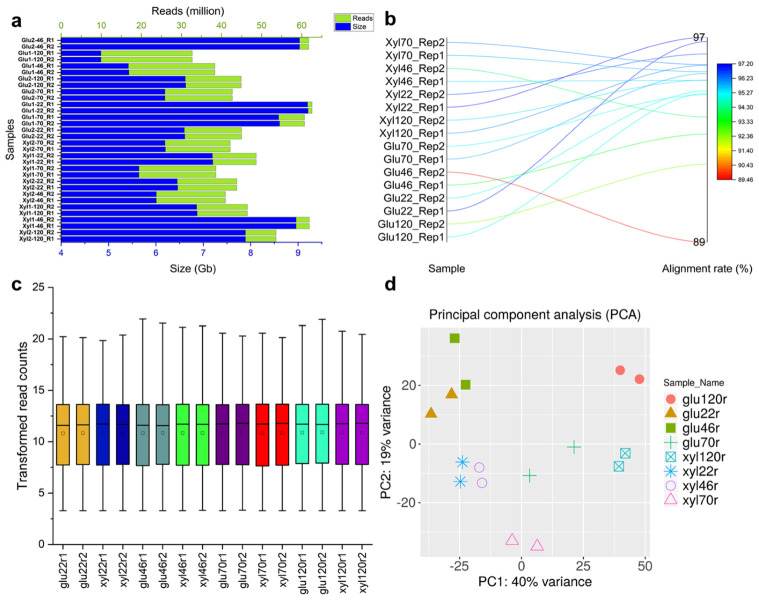
Quality metrics of RNA-sequencing reads of *Saitozyma podzolica* DSM 27192 cultivated on glucose and xylose. (**a**) Read count and sizes per library from all samples, including both strands of each read pair. (**b**) Overall alignment rate (%) on the draft genome of *S. podzolica* DSM 27192. (**c**) Boxplot of normalised and transformed read counts. (**d**) Principal component analysis plot showing variance among samples and between biological replicates.

Four RNA samples, one from the batch phase and three from the fed-batch phases, have been sequenced using NextSeq 2 × 150 (v2.5) sequencing chemistry. After trimming out sequencing adapters and filtering out low-quality reads, the data comprised ~1.5 billion high-quality and clean reads of ~224 Gb (Figure 2a). The glucose dataset comprised a range of ~32.7–62.6 million reads of size ranging between ~4.8 and ~9.2 Gb and for xylose the dataset consisted of ~38.6–61.9 million reads ranging between ~5.6 and ~9.0 Gb in size. Initial reads alignment using Hisat2 against the draft genome of *S. podzolica* DSM 27192 [20] resulted in an average read alignment rate of 95.05% with a range of ~89.47–97.19% (Figure 2b), signifying the quality and the accuracy of the sequencing data. StringTie assembly evaluation using GffCompare [25] revealed 10,670 super-loci, including 2039 (19.1%) novel loci, which represent transcripts that are missing or novel isoforms of genes in the previous annotation of *S. podzolica* DSM 27192.

To improve the downstream gene expression analysis, we removed genes with inconsistent expression by implementing a stringent filter (minimum counts per million (CPM) ≥ 0.5 in a minimum of 2 libraries), resulting in 10,506 genes with various expression profiles across the samples. The distribution of normalised gene counts based on the regularized logarithm transformation (rlog) implemented in DESeq2 [26] is presented in Figure 2c,d. Clustering of the samples, using principal component analysis (PCA), showed that biological replicates for each sampling point cluster distinctly from samples of other time points. Interestingly, samples from 22 and 46 h sampling points cluster more closely together for both glucose and xylose grown yeast compared to those from 70 and 120 h samples, suggesting greater variability between the former and the latter sets. However, the first two principal components explained only 59% of the observed variability, suggesting that differences exist among transcriptome profiles of *S. podzolica* DSM 27192 growing on glucose and xylose, and across the four sampling time points.

### 3.2. Differential Gene Expression during Initial Growth under Glucose and Xylose in DSM 27192

To investigate possible genetic differences and genes shaping initial growth of *Saitozyma podzolica* DSM 27192 on glucose and xylose, we compared gene expression profiles of the 22 h samples using DESeq2 [26]. There were 361 differentially expressed (FDR < 0.05, and log2FC > 1.5; Figure S1a) genes (representing ~3.4% of the expressed genes) observed when the yeast was grown on glucose compared to xylose. Further evaluation of the expression profiles revealed upregulation of 115 and down-regulation of 246 genes during growth on glucose relative to xylose (Figure S1b).

To gain insight into signatures of molecular activities associated with initial growth of the yeast on glucose and xylose, we performed gene ontology (GO)-based functional enrichment analysis using WebGestalt [35]. Interestingly, various GO associated with transmembrane transport is associated with the observed overexpressed genes. For instance, overrepresentation of transmembrane transport (GO:0055085; FDR = 0.07), including at least six of the 15 genes annotated as sugar (and other) transporter genes, was observed during initial growth on glucose relative to xylose (Table 1 and Table S1). By contrast, a more diverse set of genes associated with the transmembrane transporter activity of GO:0022857 was significantly overrepresented (FDR < 0.05) among upregulated genes during growth on xylose compared to glucose (Table 1 and Table S1). As with glucose upregulated genes, the majority of these (23 out of 51 genes) have been annotated as a sugar (and other) transporter gene (Table S1). Besides the predominance of these, genes of K^+^ transporter proteins in the glucose grown yeast, formate nitrite transporter, fungal trichothecene efflux pump, mitochondrial carrier, voltage-gated K channel and other general substrate transporter proteins in xylose grown cultures, were linked to the overrepresented transmembrane transport function (Table S1), suggesting a significant variation in the movement of the two sugars in *Saitozyma podzolica* DSM 27192. Overrepresentation of transmembrane transporter GOs, which indicates active transmembrane transport of a variety of substrates has been reported in many fungi [42,43]. Unlike the observed significant and specific enrichment of these GOs in glucose and xylose grown yeast observed in this study, genes associated with both GOs (GO:0022857 and GO:0055085) were reported to be up and downregulated on glucose grown *Aspergillus fumigatus* mutants [44].

### 3.3. Distinct Profiles of Putative Sugar Transporter Genes in Saitozyma Podzolica DSM 27192 Grown on Glucose and Xylose

To evaluate the potential implication of variation in the above differentially expressed sugar (and other) transporter genes during growth of the oleaginous yeast on xylose and glucose, we performed additional annotation of the predicted proteins using conserved domain database [36], the curated UniProtKB/Swiss-Prot [37] and transporter classification [38] databases as well as prediction of transmembrane helices using TMHMM Server v2.0 [39]. The analysis showed that the predicted proteins of 42 of the 65 differentially expressed transmembrane transport-associated genes under glucose and xylose (Table S1) shared significant homology (e-value, and a bit score range of 1.0 × 10^−13^–5.0 × 10^−220^ and 75.1–761, respectively) with members of sugar porter family: 2.A.1.1. However, these proteins shared low homology with the sugar porter proteins, and the curated yeast transporters, with similarity values ranging between 23.3–72.5%, respectively, suggesting novelty of the putative transporters in *S. podzolica* DSM 27192 when compared to their closest characterised relatives.

**Figure 3:**
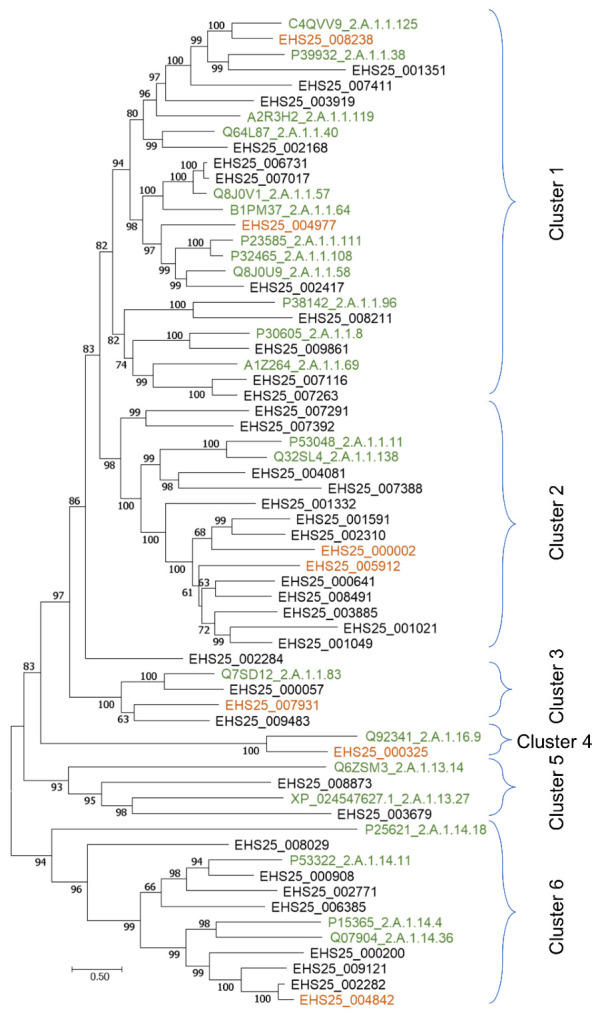
Maximum likelihood phylogeny of sugar (other) transport proteins predicted from overexpressed genes in *Saitozyma podzolica* DSM 27192 during initial growth (22 h time point) on glucose and xylose. The tree was generated using the trimmed alignment (364 amino acids) of 64 protein sequences, including 22 closest references from TCDB based on LG + F+ R6 model with -bb 1000 using IQ-TREE v1.6.11. Orange and black fonts indicate overrepresented proteins (translated genes) in glucose and xylose, respectively, while green fonts represent top matching proteins from the transporter classification database. The alignment was generated using transmembrane proteins (PSI/TM-Coffee) function of T-Coffee.

Phylogeny-based evaluation of proteins of the above sugar porter genes showed a clustering of the 7 and 35 putative sugar (and others) transporters significantly overexpressed in glucose and xylose, respectively, in six groups. (Figure 3). Cluster 1 harbours the majority of the functionally characterised sugar transporters included in this analysis and includes 2 and 11 putative transporters whose genes were significantly overexpressed in glucose and xylose, respectively. However, within this cluster, distinct transporter proteins whose genes were significantly upregulated (log2FC = 2.0 and FDR = 4.0 × 10^−2^) in glucose (EHS25_008238) and xylose (EHS25_001351; log2FC = 1.4 and FDR = 1.2 × 10^−2^) cluster with glycerol: H^+^ symporters of families 2.A.1.1.125 and 2.A.1.1.38, respectively. Orthologue of 2.A.1.1.125, characterised in *Schizosaccharomyces pombe* was shown to share regions of transmembrane domain with several other transmembrane transporters, including those of xylose, glucose and glycerol [45] and showed varied expression depending on available substrate [46], indicating that the overexpressed gene in *S. podzolica* is likely a glucose transporter. By contrast, the gene of *STL1* (2.A.1.1.38) was reported to be repressed alongside glycerol transport by glucose in *Saccharomyces cerevisiae* [47]. However, the low homology (30.3% similarity value) shared between EHS25_001351 and homologues of *STL1* suggest that the former is a putative xylose rather than glycerol transporter but similarly repressed by glucose in *S. podzolica*. This is further supported by the presence, within the same cluster, of one xylose facilitator of family 2.A.1.1.40 [48] which grouped closely with the xylose overexpressed (log2FC = 2.90 and FDR = 1.4 × 10^−13^) EHS25_002168, sharing 39% identity. In *S. cerevisiae*, homologues of *STL1* and xylose transporters share similarity values ≤ 27% [47].

The second subcluster includes glucose overexpressed (log2FC = 1.2 and FDR = 1.0 × 10^−8^) protein (EHS25_004977) distinctly clustering with families of high affinity glucose transporters of 2.A.1.1.57, low affinity glucose transporters (2.A.1.1.108) and low affinity glucose: H^+^ symporter along with one xylose overexpressed (log2FC = 1.9 and FDR = 7.6 × 10^−18^) protein (EHS25_002417) while two proteins, EHS25_006731 (log2FC = 4.7 and FDR = 5.1 × 10^−35^) and EHS25_007017 (log2FC = 3.6 and FDR = 8.7 × 10^−14^), upregulated in xylose clusters with families 2.A.1.1.57 and 2.A.1.1.64. The former includes several high affinity glucose and other monosaccharide, including xylose symporters while the latter is a family of hexose sensors [49,50]. Some of the xylose induced transporters also affiliate to the more general or broad sugar transporters such as sugar and polyol transporter 1 (2.A.1.1.69) and probable metabolite transport protein (2.A.1.1.96). Interestingly, this subcluster contains putative sugar transporter genes with the highest expression level in xylose relative to glucose grown *S. podzolica*. These include EHS25_007263 (log2FC = 9.86 and FDR = 2.1 × 10^−4^) and EHS25_009861 (log2FC = 9.34 and 1.6 × 10^−3^, Table S1) which are closely affiliated with families 2.A.1.1.69 (above) and 2.A.1.1.8 (myoinositol:H+ symporter) in the sugar (and others) transporter phylogeny.

The second major clade (Figure 3), which includes two proteins of genes, EHS25_000002 (log2FC = 1.4 and FDR = 2.7 × 10^−3^) and EHS25_005912 (log2FC = 2.3 and FDR = 1.1 × 10^−6^), upregulated in glucose and 12 proteins of xylose upregulated genes cluster distantly with proteins from two families, general α-glucoside: H^+^ symporter (2.A.1.1.11) and maltose permease (2.A.1.1.138). Combined, these proteins shared a range % identity of 23.3–35.5%, supporting their distinctiveness from the maltose transporters. Furthermore, genes coding proteins of both 2.A.1.1.11 and 2.A.1.1.138 have been reported to be repressed by glucose in *Saccharomyces pastorianus* and *Hansenula polymorpha*, respectively [51,52]. Cluster 2 harbours putative sugar transporter genes showing the highest expression levels among the analysed sugar (and others) transporter under xylose relative to glucose (Table S1). For instance, EHS25_000641, EHS25_001049, EHS25_001332, and EHS25_008491 were significantly up-regulated (FDR < 0.05) with log2FC values ranging between 9.07 and 12.69 (Table S1).

Cluster 3 also includes two putative sugar transporter genes EHS25_000057 and EHS25_009483, which were highly induced under xylose relative to glucose with log2FC values of 11.59 and 10.26 (Table S1). The proteins of these significantly overexpressed (FDR < 0.05) putative sugar transporter genes, however, grouped with a cellobiose/cellotriose/cellodextrin/lactose transporter (2.A.1.1.83; Figure 3) but as with the predicted proteins described in clusters 1 and 2, shared low similarity values ranging between 34.4 and 41.5 with the closest characterised protein of the family 2.A.1.1.83 [53], thereby suggesting a different role for the *S. podzolica* orthologues of this family.

Aside from the above three clusters, which contain putative sugar transporters, highly induced in the presence of glucose or xylose the rest of sugar (and other) proteins were grouped in clusters 4, 5 and 6 (Figure 3). Cluster 4 include a single *S. podzolica* protein with a significantly overexpressed (FDR < 0.05) gene under glucose relative to xylose and affiliated siderophore iron transporter 3, Str3 (2.A.1.16.9) in the phylogeny (Figure 3). By contrast, cluster 5 comprises two proteins whose genes were overexpressed (FDR < 0.05) under xylose relative to glucose with the proteins being the most similar MFS transporter MFSG; family 2.A.1.13.27. Str3 participates in iron homeostasis in *Schizosaccharomyces pombe* and MFSG was associated with tolerance against glucosinolates *S. cerevisiae* [54].

Cluster 6 represents the third largest cluster, comprising proteins of 7 xylose and a single glucose-induced gene (Table S1). *S. podzolica* DSM 27192 proteins included in this cluster grouped distinctly with the anion:cation symporter (2.A.1.14) family of characterised transporters associated with various substrates including monocarboxylic acids (2.A.1.13.14), dipeptides (2.A.1.14.4) and thiamine pathway *THI73* (2.A.1.14.36; Figure 3).

Combined, evidence from phylogeny, sequence homology and gene expression levels strongly suggest a putative sugar transport role for proteins included in cluster 1, 2 and 3. The cache of putative sugar transporters in *S. podzolica* DSM 27192 could be used, therefore, to enhance production of useful bioproducts from a wide range of biomass hydrolysates. However, fermentation experiments with the closely related oleaginous yeast *C. curvatus*, and other oleaginous yeasts, revealed a repression of the consumption of xylose and other sugars by glucose [51,52,55] and a decrease in consumption of both glucose and xylose by other components of biomass hydrolysate [56]. The ability of our yeast to utilize a mixture of sugars or to grow directly on the hydrolysates is still under consideration. Future characterisation of the putative *S. podzolica* sugar transporter proteins is, however, necessary to validate the predicted roles and the precise mechanisms associated with the predicted function.

### 3.4. Comparison of Transcriptome Profiles of Saitozyma Podzolica DSM 27192 during Continuous Feeding on Glucose and Xylose

**Figure 4:**
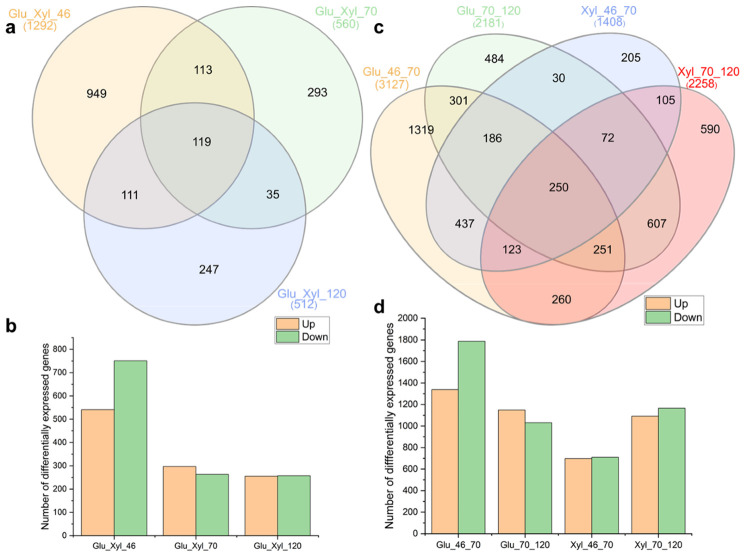
Differential gene expression (DGE) in *Saitozyma podzolica* DSM 27192 during fed batch cultivation on glucose and xylose. (**a**) Overlapping DGE for comparison between glucose and xylose grown cultures, (**b**) Number of up and down regulated genes in xylose grown cultures relative to glucose, (**c**) Overlapping DGE across the three time points in glucose and xylose grown cultures and (**d**) Number of up and downregulated genes across the three time points in glucose and xylose grown cultures.

To gain insight into the gene expression dynamics during the second phase of the cultivation (fed-batch) of *S. podzolica* DSM 27192 under glucose and xylose, two sets of comparison were performed (Figure 4). First, a comparison of the expression profiles for growth under glucose and xylose at 46 h, 70 h and 120 h, was performed. A total of 2364 genes were differentially expressed (FDR < 0.05, and log2FC > 1.5) in glucose relative to xylose, over the period of the cultivation (Figure 4a). Of these, only 119 genes were common to all time points for the respective contrast between glucose and xylose profiles. The highest number (1292) of differentially expressed genes (DGEs), which includes 541 and 751 up and down regulated genes, respectively, was observed at the onset of the fed batch process (Figure 4b).

As with the expression profile from the batch cultivation and consistent with continued addition of sugars during this phase of growth, both the upregulated and downregulated genes at time point 46 h were significantly associated (FDR < 0.05) with transmembrane activity (Table 2 and Table S2). By contrast, similar numbers of DEGs were observed at time points 70 and 120 h (Figure 4a,b). Consistently, hydrolase activity, hydrolysing O-glycosyl compounds (GO:0004553) was downregulated across the three time points in cultures grown with glucose relative to xylose (Table 2 and Table S2) as observed under batch cultivation (Table 1). Xylose induction of carbohydrate-active enzymes as well as repression of carbon catabolite by both glucose and xylose have been reported in different fungi [57,58,59]. For instance, cellulases and xylanases have been induced using xylose in *Thermoascus aurantiacus* [60]. Overexpression of these genes, therefore, suggests that xylose grown *S. podzolica* could be utilized or improved for an enhanced production of these enzymes.

Aside from these, two genes of NAD^+^-dependent formate dehydrogenase EHS25_002775 (FDR = 0.01 and log2FC −3.01) and EHS25_003099 (FDR = 0.02 and log2FC −1.78) were significantly downregulated in glucose relative to xylose grown cultures at 46 h (Table S2). Formate dehydrogenase oxidises formate to CO_2_ and the NADH generated from the reaction may serve as a source of reducing equivalents [61,62]. Formate dehydrogenase activity has been reported in *S. cerevisiae* and *Candida tropicalis* grown on glucose and xylose, respectively, as well as in a combination of these sugars with formate [61,62]. Two possible sources of formate during fermentation of glucose or xylose in *S. podzolica* are either exogenous formate in the culture medium or formate produced via NAD/NADP—NADH/NADPH interconversion and other pathways [63], with the latter source being more likely. Downregulation of formate dehydrogenase genes, EHS25_002530 (log2FC = −4.79 and FDR = 2.33 × 10^−3^), EHS25_002775 (log2FC = −3.92 and FDR = 1.7 × 10^−6^) and EHS25_003099 (log2FC= -3.28 and FDR = 9.19 × 10^−11^) at 46 relative to 70 h sampling point and upregulation of EHS25_003099 (log2FC = 1.33 and FDR =2.04 × 10^−2^) at 70 relative to 120 h sampling point were also observed in culture growing under glucose (Table S2), suggesting significant formate dehydrogenase activity in glucose grown *S. podzolica*. Further evaluation of data revealed a significant upregulation of 3,4-dihydroxy-2-butanone 4-phosphate synthase gene (EHS25_006321; log2FC = 0.72 and FDR = 4.07 × 10^−2^) at 46 relative to 70 h in glucose grown cultures (Table S2), suggesting a greater production of formate in glucose grown *S. podzolica*. 3,4-dihydroxy-2-butanone 4-phosphate synthase is a key enzyme of flavin biosynthesis catalysing the conversion of d-ribulose 5-phosphate to 1-deoxy-l-glycero-tetrulose 4-phosphate by releasing formate [64].

Gene ontology-based enrichment analysis further revealed significant downregulation of genes associated with cellular respiration (GO:0045333: FDR = 7.4 × 10^−3^) at 46 h sampling point and cellular carbohydrate metabolic process (GO:0044262; FDR = 3.6 × 10^−2^) at 70 h sampling point in *S. podzolica* grown on glucose relative to xylose (Table 2). However, a more comprehensive comparison of carbohydrate metabolism under the two sugars is presented below. Glycolysis, Pentose Phosphate, and Glucuronate Interconversion Pathways and TCA Cycle during Initial Growth of Saitozyma Podzolica DSM 27192 on Glucose Relative to Xylose

**Figure 5:**
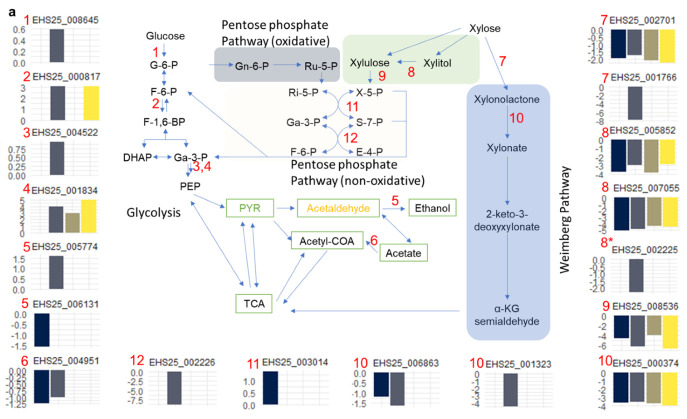

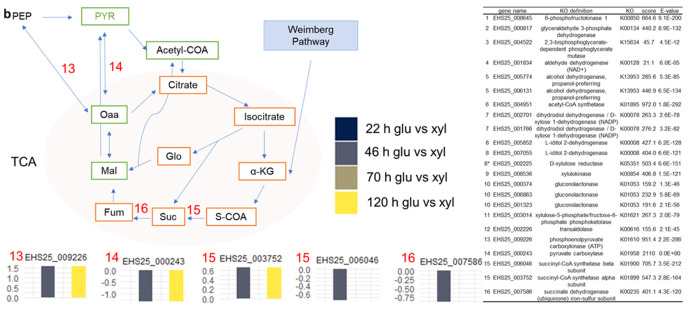
Overview of expression pattern of genes associated with enzymes of the central carbon metabolism of *Saitozyma podzolica* DSM 27192 on glucose relative to xylose. (**a**) glycolysis, pentose phosphate and putative Weimberg pathways. (**b**) TCA cycle. Differentially expressed genes (FDR < 0.05) are indicated by red numbers in the pathways and bar plots of log2FC values for glucose relative to xylose linked to these numbers. Green and light brown boxes indicate pathways that occur in both cytoplasm and mitochondria, and those occurring exclusively in the mitochondrion, respectively. Figure was adapted from [68]. Asterisk ‘*’ indicates that d-xylulose reductase is also called xylitol dehydrogenase.

To further elucidate the functional difference between transcriptome profiles of glucose and xylose grown *S. podzolica*, we, in addition to gene ontology, scanned relevant metabolic pathways associated with the overexpressed genes at 22 h time point using against KEGG ortholog [32]. Using Pathview [65], KO identities of the differentially expressed genes were mapped against several important pathways (Figure 5a,b). No significant difference was observed for the comparison of TCA cycle between the two transcriptome profiles during the initial growth of the yeast (time point 22 h: Figure 5b). By contrast, two genes each of glycolysis and pentose phosphate pathway show significant differences between the two conditions. In glycolysis, EHS25_004951 (log2FC = 1.2 and FDR = −5.0 × 10^−4^) and EHS25_006131 (log2FC = −1.6 and FDR = 2.0 × 10^−2^) coding acetyl-CoA synthetase (EC:6.2.1.1) and alcohol dehydrogenase, propanol-preferring (EC:1.1.1.1), respectively, were significantly induced in xylose relative to glucose condition (Figure 5a). The former catalyses the activation of acetate to acetyl-CoA while the latter catalyses the first step of alcohol degradation. Previous studies in *S. cerevisiae* [66] showed that the expression of acetyl-CoA synthetase gene is strongly suppressed under carbon source limitation and high glucose concentration. Contrary to our observation, however, no significant difference exists in expression of the gene between recombinant xylose-utilizing *S. cerevisiae* grown on glucose and xylose [67]. Our observation, suppression, and overexpression of the alcohol dehydrogenase gene under glucose and xylose, respectively, was reported in these studies [66,67]. The overexpression of acetyl-CoA synthetase gene under xylose could prove significant for developing fermentation strategies on different C-sources in *S. podzolica*.

Of the fifteen genes associated with pentose phosphate and glucuronate interconversions, glycolysis pathways and TCA cycle, only two genes have been overexpressed in glucose relative xylose grown yeast over the duration of the batch fed process (Figure 5). By contrast, five genes of the pentose and glucuronate interconversion and two of pentose phosphate pathways were significantly suppressed under glucose compared to growth under xylose. As expected, significant overrepresentation of genes encoding d-xylulose reductase/l-iditol 2-dehydrogenase (EC:1.1.1.9/EC:1.1.1.14), EHS25_007055 (log2FC = −5.1 and FDR = 2.0 × 10^−51^) and EHS25_005852 (log2FC = −3.8 and FDR = 4.0 × 10^−31^), which catalyses the oxidation of xylitol to d-xylulose [69] was observed in xylose relative to glucose (Figure 5a). Xylulokinase (EC:2.7.1.17), EHS25_008536 (log2FC = −4.7 and FDR = 5.0 × 10^−37^), which catalyses xylulose to xylulose-5-phosphate phosphorylation was also overrepresented under xylose.

Comparison of the transcriptome profiles also revealed three genes linked to pentose phosphate pathway differing significantly between glucose and xylose grown *S. podzolica* (Figure 5a). The putative phosphoketolase (EC:4.1.2.9/4.1.2.22), encoded by EHS25_003014 in *S. podzolica* is significantly overrepresented (log2FC = 1.4 and FDR = 9.6 × 10^−5^) under glucose. However, induction of this gene under glucose suggests an acetate production pathway, where the putative phosphoketolase 4.1.2.22, catalyses the cleavage of xylulose-5-phosphate to acetyl phosphate and erythrose 4-phosphate as demonstrated in *Cryptococcus neoformans* [70]. However, the present data showed that the gene encoding acetate kinase, which converts acetyl-phosphate from the previous reaction to acetate [70,71,72], was similarly expressed in both glucose and xylose grown *S. podzolica*. Putative genes encoding glucose oxidase, which catalyse the oxidation of d-glucose to d-glucono-1,5-lactone, and d-xylose to d-Xylono-1,5-lactone [73,74], showed similar expression values under glucose and xylose. However, two putative gluconolactonase [EC:3.1.1.17] genes, EHS25_006863 (log2FC = −1.2 and FDR = 1.0 × 10^−4^) and EHS25_000374 (log2FC = −3.7 & FDR = 1.4 × 10^−65^), showed greater level of expression under xylose (Figure 5a). Gluconolactonase [EC:3.1.1.17] catalyses the conversion of d-glucono-1,5-lactone to d-gluconate [73]. Overrepresentation of the two genes in the xylose grown yeast may indicate expression of xylonoactonase [EC:3.1.1.68] associated with Weimberg pathway [75,76]. This is supported by the overexpression of EHS25_002701 (log2FC = −1.9 and FDR = 1.64 × 10^−14^) coding dihydrodiol dehydrogenase/d-xylose 1-dehydrogenase (NADP) (EC:1.3.1.20 1.1.1.179) under xylose growth and accumulation of xylonic acid reported in [10]. These expression profiles provide the essential information for future exploration of this pathway in developing or engineering *S. podzolica* with efficient xylose utilization capabilities.

Comparison of the gene expression during fed-batch cultivation for the individual sugars revealed that 5308 and 3666 genes were differentially expressed (FDR < 0.05) across the three time points in glucose and xylose, respectively (Figure 4c). Of these 8,974 genes that were differentially expressed in both sugars, ~2.8% (250) were observed among all time points and both sugars (Figure 4c). Higher numbers of genes (3217), comprising 1339 and 1788 up and downregulated genes, were observed to be differentially expressed at 46 relative to the 70 h time point in glucose grown cultures compared to those grown on xylose (Figure 4d). By contrast, a greater number of genes (2258), comprising 1092 and 1166 genes were significantly (FDR < 0.05) up and downregulated, respectively, at 70 relative to 120 h time point in xylose compared to glucose grown cultures. Evaluation of the functional basis of the DEGs for the various profile comparisons, however, showed different metabolic patterns during growth of the oleaginous yeast on glucose and xylose (Table 2). For instance, at 46 relative to 70 h time point, 115 genes associated with the carboxylic acid metabolic processes (FDR = 8.7 × 10^−6^) were downregulated under glucose compared to 9 genes of acyl-CoA metabolic process (FDR = 3.1 × 10^−2^) for the same time point in xylose (Table 2).

**Figure 6:**
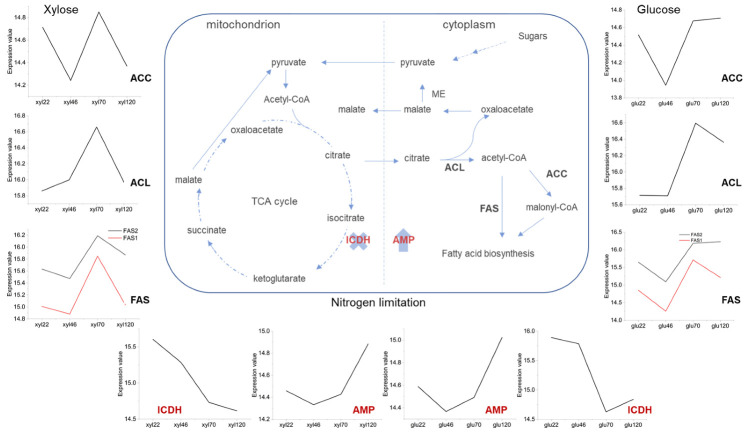
Profiles of major genes linked to initiation and production of single cell oil during growth of *Saitozyma podzolica* DSM 27192 on glucose and xylose under nitrogen limitation. ACC, ACL, AMP, FAS and ICDH refer to acetyl-CoA carboxylase, ATP citrate lyase, AMP deaminase, fatty acid synthase and isocitrate dehydrogenase respectively. Line graphs show expression trajectories of genes encoding ACC, ACL, AMP, FAS and ICDH. X and arrow up indicate suppression of ICDH and overexpression of AMP.

Interestingly, among genes linked to induction of oil production (Figure 6), only ATP citrate lyase (ACL) gene showed significant over expression in both glucose (EHS25_008599; log2FC = 1.20 and FDR = 5.4 × 10^−4^) and xylose (EHS25_008599; log2FC = 0.92 and FDR = 2.11 × 10^−2^) at 70-h time point. ACL catalyses the cleavage of citrate into acetyl-CoA and oxaloacetate (OAA). Preference to this pathway as well as the cycling of carbon (OAA → pyruvate → citrate; Figure 6) is characteristic of oleaginous yeast [77,78]. Genes of acetyl-CoA carboxylase (ACC), fatty acid synthase subunit alpha (FASI; EHS25_002838; log2FC = 1.92 and FDR = 5.35 × 10^−5^) & beta (FASII; EHS25_002837; log2FC = −1.58 and FDR = 8.00 × 10^−10^) and isocitrate dehydrogenase subunit 1 & 2 (IDH1 & 2) were significantly overexpressed (FDR > 0.05) at 70 relative to 46 h time point (Figure 6) only in glucose grown cultures (Table S2). The above genes were also upregulated in xylose grown cultures, but outside the significance threshold of this analysis. ACC catalyses the carboxylation of acetyl-CoA (A-CoA) to malonyl-CoA (M-CoA) while FAS complex catalyses fatty acid synthesis through a series of reactions (condensation—reduction—dehydration), involving A-CoA, M-CoA, and NADPH [79]. However, greater suppression of isocitrate dehydrogenase (ICDH) gene (EHS25_006623) was observed in glucose (log2FC = −1.7 and FDR = 4.37 × 10^−9^) relative to xylose (log2FC = −0.79 and FDR = 2.4 × 10^−2^) grown cultures at 46- relative to 70-h sampling point. By contrast, AMP deaminase (AMPD) gene (EHS25_008352) was not significantly overexpressed under both sugars. The mechanism of oil production initiation in oleaginous yeast has been variously linked to nutrient limitation, including nitrogen [80,81,82]. Nitrogen starvation induces an increase in AMPD activity leading to depletion of adenosine monophosphate (AMP) thereby inhibiting ICDH and as a consequence the accumulation of citrate [80].

Aside from the dynamics of AMPD and ICDH above, several studies have reported the expression profiles of the ‘oleagenic’ genes in yeast during oil accumulation under various conditions. For instance, in *Trichosporon oleaginosus* grown under two carbon sources and nitrogen or phosphate limitations, only ACL and FAS genes were significantly overrepresented (FDR < 0.05) in the yeast grown in xylose under nitrogen limitation relative to the control cultures and only ACL gene significantly overrepresented (FDR < 0.05) in N-acetyl glucosamine grown yeast under nitrogen limitation relative to the control cultures [83]. By contrast, the current study did not reveal significant differences in the expression of these genes in glucose compared to xylose grown *S. podzolica* under nitrogen limitation (sampling time 76 h; Figure 1). Apparently, the observed overexpression (FDR < 0.05) of the ACL gene (also reported in [83]) and suppression (FDR < 0.05) of the ICDH gene likely underpin lipid accumulation under nitrogen limitation in *S. podzolica*.

### 3.5. Temporal Dynamics of S. podzolica DSM 27192 Genes Grown under Glucose and Xylose

**Figure 7:**
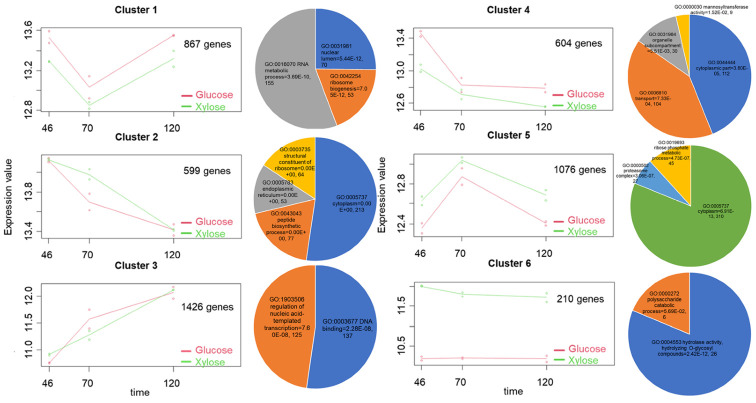
Trajectories and GO functional enrichment of expressed gene clusters in *Saitozyma podzolica* DSM 27192 cultivated glucose and xylose. Profiles of six clusters over three time points (46, 70 and 120 h) were generated using maSigPro and gene ontology-based functional association was plotted in the adjacent pie charts. In the line graphs, the dots per time point represent the mean expression values per biological replicate. Gene ontology (GO), false discovery rate values and number of genes associated with each GO are shown in the pie charts.

To identify the gene expression profile changes over the duration of *S. podzolica* DSM 27192 growth on glucose and xylose under fed-batch condition (46, 70 and 120 h), we implemented the maSigPro algorithm based on alfa threshold value of 0.5. The analysis revealed 4782 genes (~47% of the expressed genes) showing significant differential expression (FDR < 0.05) during the continuous cultivation experiment. The co-expressed genes were partitioned based on the *K*-means cluster method (*k* = 6 and *R*^2^ ≥ 0.7) into six distinct expression clusters (Figure 7). Clusters 1 and 5, comprising 867 (18.13% of the co-expressed genes) and 1076 (22.5% of the co-expressed genes) genes, respectively, show opposite trajectories, with expression values in the former cluster decreasing at 70 h before then peaking again at 120 h. Evaluation of genes in cluster 1 revealed an overrepresentation (FDR < 0.05) of two biological processes (BP), ribosome biogenesis, (GO:0042254) and RNA metabolic process (GO:0016070), as well as one cellular component (CC) function, nuclear lumen (GO:0031981), suggesting a possible suppression of translational machinery in response to nitrogen limitation. Previous studies have reported the influence of nutrients availability, including nitrogen, on ribosome biogenesis [84,85,86]. Specifically, proteome study of the oleaginous yeast *Rhodotorula toruloides* grown in glucose and xylose revealed significant depletion of genes associated with ribosome biogenesis [87].

By contrast, two CC functions, cytoplasm (GO:0005737) and proteasome complex (GO:0000502; also included in GO:0005737) and one BP, ribose phosphate metabolic process (GO:0019693; also included in GO:0005737) were significantly overrepresented (FDR < 0.05) among genes belonging to cluster 5 (Figure 7). Upregulation of these processes provides insight into the innate evolutionary strategy adapted by the yeast to salvage the effects of nitrogen-starvation-induced stress. For instance, overrepresentation of the proteasome complex indicates increased destructive activity of the proteasome system on cyclin, which prevents organelle dismemberment and cell destruction under nitrogen limitation [88]. The potential of cyclin activity is also indicated by the upregulation of genes of cyclin (EHS25_000231) and cyclin binding protein (EHS25_007811), included among genes associated with cytoplasmic function (Figure 7; Table S3). In addition, the increased expression of genes linked to energy production and other metabolic pathways (Table S3) under complete nitrogen starvation indicates robust metabolic capacity of the yeast at the onset of lipid accumulation.

Similar reverse trajectories were also observed between clusters 2 (599 genes; 12.5% of the co-expressed genes) and 3 (1426 genes; 29.8% of the co-expressed genes) showing trends of decrease and increase, respectively, during growth of *S. podzolica* DSM 27192 on glucose and xylose. Cluster 2 is significantly (FDR < 0.05) enriched in two CC, one BP and an MF, structural constituent of ribosome (GO:0003735), while cluster 3 shows significant overrepresentation (FDR < 0.05) in genes linked to one BP (GO:1903506; regulation of nucleic acid-templated transcription) and one MF (GO:0003677; DNA binding; Figure 7). As discussed above, nitrogen limitation induces the downregulation of ribosome biogenesis. However, in contrast to the trajectory of cluster 1, the depletion of the structural constituent of ribosome and peptide biosynthetic process (GO:0043043) persisted in cluster 2, corresponding to the phase of maximum oil accumulation (72–120-h time point) in *S. podzolica* DSM 27192 [10]. Although not conclusive at present, our study indicates a clearer association of lipogenesis and downregulation of ribosomes, contrary to the suggestion of greater association with growth activity in *R. toruloides* [87]. This hypothesis is further supported by various ‘omics’ studies under nitrogen limitation in *Yarrowia lipolytica* [89,90].

Cluster 4 with 604 (12.6%) genes showed a similar rapid drop in expression values as with cluster 1 at time point 70, but the expression levels remained low through to time point 120 h in both glucose and xylose cultures (Figure 7). Evaluation of the temporal dynamics represented in this cluster revealed a predominance of genes linked to mannosyltransferase activity (GO:0000030), organelle compartment (GO:0031984), transport and cytoplasm GOs with depletion of nitrogen and the onset of lipogenesis (Figure 7). Specifically, there was a decrease in the expression of several key genes linked to protein *N*-glycosylation [91,92,93] such as the putative α-1,2-, α-1,3-, α-1,6-, and β-1,4-mannosyltransferases under nitrogen limitation in cultures grown on both glucose and xylose (Table S3). Apparently, these genes are involved in Glycosylphosphatidylinositol (GPI) anchoring as suggested by a corresponding reduction in the expression of genes propagating the enriched organelle compartment, transport, and cytoplasm GOs. For instance, genes of phosphatidylinositol-glycan biosynthesis S protein (EHS25_000085; Table S3), a key component of GPI transamidase complex [94], transport protein bet1 (EHS25_004488) and endoplasmic reticulum (ER)-Golgi intermediate compartment (ERGIC) protein (EHS25_004458), both involved in shuttle between endoplasmic reticulum and Golgi membrane [95,96] further support the downregulation of ER-to-Golgi activities during nitrogen-limited lipid accumulation. However, the precise implication of these findings is subject to further investigation.

Finally, cluster 6 (210 genes; 4.4%) exhibited uniform expression values across the three time points in both sugars (Figure 7). However, higher gene expression values were observed for culture grown on xylose relative to glucose, consistent with the observed significant upregulation of hydrolase activity, hydrolysing O-glycosyl compounds (GO:0004553) throughout the cultivation period in xylose compared to glucose grown cultures (discussed above; Table 1 and Table 2).

## 4. Conclusions

Previous studies have reported the ability of *S. podzolica* to produce various biochemicals, including single-cell oils and sugar acids from different carbon sources [10]. Thus, we evaluated the transcriptome profiles in *Saitozyma podzolica* DSM 27192 cultivated on glucose and xylose under batch and continuous processes by RNA-seq analysis. Our transcriptomic analysis revealed a wide range of transcriptome level differences between *S. podzolica* grown on glucose and xylose. We showed that several putative sugar transporter genes, including transporters of other substrates, were overexpressed distinctively on either glucose or xylose, albeit in greater numbers in the latter. This study also reports xylose induction of genes associated with carbohydrate utilization, a feature which could be harnessed potentially for enhanced cultivation of the yeast on complex substrates. We also discussed the presence of putative genes associated with the initial steps of the Weimberg pathway, which likely underpin the efficient utilization of xylose and production of xylonic acid by *S. podzolica*. However, the basis of carbon flux partitioning between the above pathway and the non-oxidative pentose phosphate pathway remains unclear, given that genes coding for enzymes of both pathways were significantly expressed under xylose cultivation. Our study contributes to a better understanding of the genetic basis of glucose and xylose growth and production of single cell oil by *S. podzolica*. These data will contribute to further development of efficient fermentation strategies and development of *S. podzolica* strains with superior features for enhanced biotechnological applications.

## Figures and Tables

**Table 1:** Functional enrichment of differentially expressed genes (at 22 h time point) of *Saitozyma podzolica* DSM 27192 growing on glucose compared to xylose based on WebGestalt. BP and MF indicate biological process and molecular function, respectively. DE and FDR represent differential expression and false discovery rate, respectively.

DE	Category	GO	Ontology Definition	Size	Overlap	Expect	Ratio	*p*-Value	FDR
Up	BP	GO:0055085	transmembrane transport	725	15	5.08	2.95	4.4 × 10^−5^	7.1 × 10^−2^
	MF	GO:0022857	transmembrane transporter activity	650	14	4.55	3.07	5.8 × 10^−5^	7.1 × 10^−2^
	MF	GO:0005215	transporter activity	660	14	4.62	3.03	6.9 × 10^−5^	7.1 × 10^−2^
Down	MF	GO:0022857	transmembrane transporter activity	650	51	20.69	2.46	1.8 × 10^−10^	4.7 × 10^−7^
	MF	GO:0005215	transporter activity	660	51	21.01	2.43	3.1 × 10^−10^	4.7 × 10^−7^
	MF	GO:0004553	hydrolase activity, hydrolyzing O-glycosyl compounds	171	24	5.44	4.41	4.5 × 10^−10^	4.7 × 10^−7^
	BP	GO:0055085	transmembrane transport	725	53	23.08	2.30	9.7 × 10^−10^	7.5 × 10^−7^
	MF	GO:0016798	hydrolase activity, acting on glycosyl bonds	184	24	5.86	4.10	2.1 × 10^−9^	1.3 × 10^−6^
	BP	GO:0005975	carbohydrate metabolic process	357	33	11.36	2.90	1.3 × 10^−8^	6.7 × 10^−6^
	BP	GO:0016052	carbohydrate catabolic process	89	11	2.83	3.88	1.0 × 10^−4^	4.5 × 10^−2^
	BP	GO:0000272	polysaccharide catabolic process	37	7	1.18	5.94	1.3 × 10^−4^	5.2 × 10^−2^
	MF	GO:0004181	metallocarboxypeptidase activity	10	4	0.32	12.57	1.8 × 10^−4^	6.1 × 10^−2^

**Table 2:** Functional enrichment of differentially expressed genes in *Saitozyma podzolica* DSM 27192 grown on glucose compared to xylose during fed batch cultivation based on WebGestalt. BP, CC and MF represent biological process, cellecular component and molecular function, respectively. DE and FDR represent differential expression and false discovery rate, respectively.

Comparison	DE	Function	GO	Definition	Size	Overlap	Expect	Ratio	*p*-Value	FDR
Glu-Xyl-46	Up	MF	GO:0022857	transmembrane transporter activity	685	50	26.07	1.92	2.1 × 10^−6^	5.0 × 10^−3^
		MF	GO:0046915	transition metal ion transmembrane transporter activity	21	7	0.80	8.76	7.7 × 10^−6^	7.0 × 10^−3^
	Down	MF	GO:0004553	hydrolase activity, hydrolyzing O-glycosyl compounds	179	40	13.21	3.03	8.2 × 10^−11^	2.6 × 10^−7^
		MF	GO:0022857	transmembrane transporter activity	685	87	50.53	1.72	7.1 × 10^−8^	4.4 × 10^−5^
		BP	GO:0045333	cellular respiration	48	13	3.54	3.67	2.8 × 10^−5^	7.4 × 10^−3^
Glu-Xyl-70	Down	MF	GO:0004553	hydrolase activity, hydrolyzing O-glycosyl compounds	179	28	4.65	6.03	4.0 × 10^−15^	1.2 × 10^−11^
		BP	GO:0010383	cell wall polysaccharide metabolic process	26	6	0.67	8.89	4.1 × 10^−5^	2.6 × 10^−2^
		BP	GO:0044262	cellular carbohydrate metabolic process	106	11	2.75	4.00	8.1 × 10^−5^	3.6 × 10^−2^
Glu-Xyl-120	Down	MF	GO:0004553	hydrolase activity, hydrolyzing O-glycosyl compounds	179	21	4.54	4.62	2.6 × 10^−9^	8.0 × 10^−6^
Glu-46-70	Up	CC	GO:0044446	intracellular organelle part	1187	220	152.43	1.44	7.3 × 10^−11^	2.3 × 10^−7^
		BP	GO:0006364	rRNA processing	136	43	17.46	2.46	5.6 × 10^−9^	5.8 × 10^−6^
	Down	BP	GO:0019752	carboxylic acid metabolic process	371	115	70.45	1.63	5.3 × 10^−9^	8.7 × 10^−6^
		BP	GO:0046395	carboxylic acid catabolic process	83	36	15.76	2.28	2.4 × 10^−7^	1.2 × 10^−4^
		BP	GO:1901565	organonitrogen compound catabolic process	230	75	43.68	1.72	3.3 × 10^−7^	1.3 × 10^−4^
Glu-70-120	Up	CC	GO:0044444	cytoplasmic part	1220	260	156.19	1.66	0	0
		BP	GO:1901564	organonitrogen compound metabolic process	1486	279	190.25	1.47	1.6 × 10^−15^	1.6 × 10^−12^
		CC	GO:0044429	mitochondrial part	205	67	26.25	2.55	3.2 × 10^−14^	2.0 × 10^−11^
	Down	CC	GO:0005634	nucleus	1293	217	135.76	1.60	2.2 × 10^−16^	6.9 × 10^−13^
		BP	GO:0051171	regulation of nitrogen compound metabolic process	665	113	69.82	1.62	2.5 × 10^−8^	1.1 × 10^−5^
Xyl-46-70	Up	CC	GO:1990904	ribonucleoprotein complex	380	76	27.29	2.78	0	0
		BP	GO:0042254	ribosome biogenesis	189	60	13.57	4.42	0	0
		BP	GO:0016072	rRNA metabolic process	153	44	10.99	4.00	2.2 × 10^−16^	8.6 × 10^−14^
	Down	BP	GO:1901565	organonitrogen compound catabolic process	230	41	19.57	2.10	2.8 × 10^−6^	8.8 × 10^−3^
		MF	GO:0048038	quinone binding	13	7	1.11	6.33	3.4 × 10^−5^	3.1 × 10^−2^
		BP	GO:0006637	acyl-CoA metabolic process	23	9	1.96	4.60	6.0 × 10^−5^	3.1 × 10^−2^
Xyl-70-120	Up	CC	GO:0005737	cytoplasm	1677	296	198.99	1.49	0	0
		BP	GO:0009150	purine ribonucleotide metabolic process	106	39	12.58	3.10	1.7 × 10^−11^	1.8 × 10^−8^
	Down	BP	GO:0042254	ribosome biogenesis	189	53	20.88	2.54	3.9 × 10^−11^	4.0 × 10^−8^
		BP	GO:0016070	RNA metabolic process	1029	173	113.66	1.52	2.0 × 10^−10^	1.5 × 10^−7^

## Data Availability

RNA-seq data generated and reported in this study have been deposited into NCBI Sequence Read Archive (SRA) database under the BioProject ID: PRJNA755891.

## Supplementary Materials

The following are available online at https://www.mdpi.com/article/10.3390/jof7090758/s1, Figure S1: Differential gene expression during initial growth (22 h time point) of *Saitozyma podzolica* DSM 27192 on glucose compared to xylose, Table S1: Differentially expressed transporter genes in *Saitozyma podzolica* DSM 27192 grown on glucose compared to xylose, Table S2: Functional enrichment of differentially expressed genes during continuous cultivation of *Saitozyma podzolica* DSM 27192 on glucose compared to xylose, Table S3: GO functional enrichment of expressed gene clusters in *Saitozyma podzolica* DSM 27192 grown on glucose and xylose.
