# Supplementary material for: Global Transcriptome Profile of the Oleaginous Yeast Saitozyma podzolica DSM 27192 Cultivated in Glucose and Xylose
Source: J Fungi (Basel). 2021 Sep 15;7(9):758. doi: 10.3390/jof7090758 (PMC8466774; doi:10.3390/jof7090758)
Supplement: Supplementary file 1 [file jof-07-00758-s001.zip › jof-1367483-figure S1.pdf]

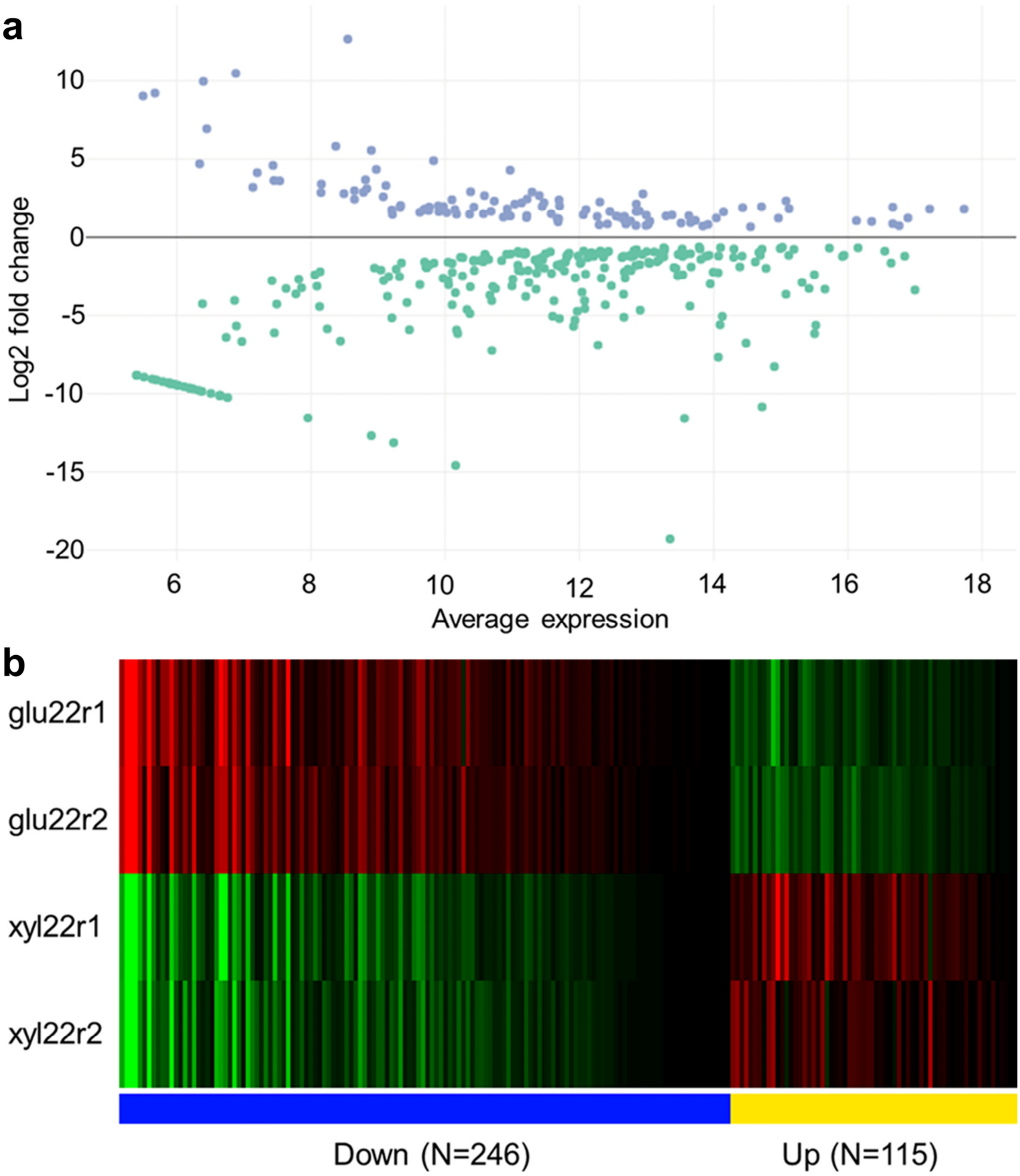

Figure S1. Differential gene expression during initial growth (22 h time point) of *Saitozyma podzolica* DSM 27192 on glucose compared to xylose. (**a**) MA plot showing log fold change (log_2FC) relative to mean gene expression values. (**b**) Heatmap showing pattern and number of differentially expressed genes title.
